# Impact of an Antimicrobial Stewardship Intervention on the Appropriateness of Carbapenem Use at a Tertiary Hospital in Malaysia

**DOI:** 10.7759/cureus.31660

**Published:** 2022-11-18

**Authors:** Chee Yik Chang, Neelam Devi Nath, Karin Lam, Masliza Zaid

**Affiliations:** 1 Infectious Diseases, Hospital Sultanah Aminah, Johor Bahru, MYS; 2 Internal Medicine, Hospital Sultanah Aminah, Johor Bahru, MYS

**Keywords:** appropriateness, antimicrobial resistance, multidrug-resistant, antimicrobial stewardship, carbapenem

## Abstract

Introduction

Carbapenems are broad-spectrum antibiotics used to treat infections caused by multidrug-resistant organisms. The inappropriate use of carbapenem results in the development of antimicrobial resistance. The aim of this study was to determine the impact of an antimicrobial stewardship intervention on the appropriateness of carbapenem use.

Method

The study includes a baseline/pre-intervention audit, an intervention, and a post-intervention study to examine the impact of antimicrobial stewardship activities on carbapenem use. Audit and data collection were carried out by filling up an audit form. The indicator for this study will be the appropriateness of carbapenem use (appropriate, suboptimal, or inappropriate) pre- and post-intervention.

Results

The overall appropriateness of carbapenem prescription in the medical wards increased from 65% to 83.3% following the implementation of antimicrobial stewardship intervention. The most common indication of carbapenem usage in our study was hospital-acquired pneumonia/ventilator-associated pneumonia.

Conclusion

Antimicrobial stewardship intervention is effective in improving the appropriateness of carbapenem use. Tailored stewardship programs are required to better control carbapenem use and combat antimicrobial resistance.

## Introduction

Carbapenems are a class of broad-spectrum antibiotics that are used to treat multidrug-resistant (MDR) organisms or as empirical therapy in severe, life-threatening infections [1]. Carbapenems have broad antibacterial activity and are effective against both Gram-positive and Gram-negative bacteria. Carbapenems are essential components of our antibiotic armamentarium, but the recent emergence of MDR pathogens has posed a serious threat to this class of life-saving antimicrobials [2].

Carbapenem resistance in Gram-negative bacteria has been a major clinical and public health problem globally. The incidence of carbapenem-resistant *Enterobacteriaceae* (CRE) was estimated to be 2.93 per 100,000 population in the United States. These MDR organisms can cause infections with high mortality and limited treatment options. Furthermore, they are becoming increasingly recognized as a significant source of healthcare-associated infections [3]. Given the association between antimicrobial use and the selection of resistant pathogens, the frequency of inappropriate antimicrobial use is often applied as a surrogate marker for the avoidable impact on antimicrobial resistance [4]. In addition, the amount of carbapenem usage also strongly correlates with the percentage of CRE isolates [5].

The implementation of an antimicrobial stewardship program is a critical component for healthcare institutions in gatekeeping judicious antimicrobial use. Antimicrobial stewardship is essential in optimizing the use of antimicrobials to prevent the development of antimicrobial resistance and improve patient outcomes. The objectives of antimicrobial stewardship include collaborating with healthcare professionals to choose the most appropriate antimicrobial for patients, along with the proper duration and dose. In addition, antimicrobial stewardship reduces the emergence of resistance by preventing the misuse, abuse, and overuse of antibiotics [6].

The objectives of this study were to evaluate the appropriateness of carbapenem orders in the medical wards of a tertiary referral hospital and to identify any differences in the level of carbapenem usage appropriateness pre- and post-antimicrobial stewardship intervention. The indications and the site of infection for patients receiving carbapenem have also been identified.

## Materials and methods

Study design

We conducted a baseline/pre-intervention audit, intervention, and post-intervention study to examine the impact of antimicrobial stewardship activities on the proper use of carbapenem. It was based on the "Plan", "Do", "Study," and "Act" (PDSA) cycle, in which testing a change entails developing a plan to test the change, executing the said test, observing and learning from the consequences, and determining necessary modifications to the test.

Phase 1: Audit the Appropriateness of Carbapenem Usage at the Baseline

The first phase involved conducting an audit on patients who were started on carbapenem in the medical ward. The patient's demographic information, medical history, choice of carbapenem, documented indications, and sites of infection were collected. The use of carbapenem was categorized as appropriate, suboptimal, or inappropriate.

Phase 2: Intervention Program

The second phase of the project consists of interventions as part of antimicrobial stewardship activities to improve the level of appropriateness of carbapenem use among the prescribers. Interventions included training for the clinicians via continuous medical education (CME) and direct feedback to the clinicians if the carbapenem order was suboptimal or inappropriate. CME includes a series of face-to-face/online lectures on antimicrobial use and resistance, as well as the promotion of national antibiotic guidelines.

Phase 3: Post-intervention Audit and Data Analysis

The third phase of the project was a post-intervention audit on the appropriateness of carbapenem usage, followed by data analysis. Data from pre- and post-intervention were compiled and compared to ascertain any positive impact of antimicrobial stewardship activities on the appropriate use of carbapenem in the medical wards.

Study Setting and Population

Hospital Sultanah Aminah (HSA) is a public, multi-specialty hospital located in Johor Bahru, Johor, Malaysia. It is the largest hospital in Johor and the main referral and tertiary health center for the state. The study population was adult patients (aged 18 years and above) admitted to the medical wards at HSA. Patients who were prescribed carbapenem were included in the study. The number of subjects enrolled was dependent on the number of subjects admitted to the wards during the audit period.

Variables

Variables included the appropriateness of carbapenem use (appropriate, suboptimal, or inappropriate), specific indications (definitive/empirical) for which carbapenem was prescribed, sites of infection, and history of recent antimicrobial exposure.

Data resource and measurement

Data Collection Tool

Audit and data collection were carried out by filling up an audit form. The indicator for this study was the appropriateness of carbapenem use (appropriate, suboptimal, or inappropriate) pre- and post-intervention. The appropriateness of carbapenem use was categorized into appropriate, suboptimal, or inappropriate, and these criteria were determined by an evaluation of carbapenem that was previously published, as shown below [7].

Appropriate: Septic shock or severe infection with a history of infection in the past six months with a β-lactam-resistant organism; septic shock or severe infection with a history of multiple antibiotics given or colonization with a β-lactam-resistant organism; severe neutropenia (absolute neutrophil count < 500/mL) with a history of multiple antibiotics given or colonization with a β-lactam-resistant organism; concern for necrotizing pancreatitis; persistent fever or hemodynamic instability in a patient who is already receiving broad-spectrum β-lactam; severe infection in a patient who has received broad-spectrum β-lactam therapy for a five-day course in the past 30 days; or as recommended or approved by an infectious disease consult.

Suboptimal: Severe sepsis of unknown cause; healthcare-associated pneumonia; severe intra-abdominal infection; severe neutropenia (absolute neutrophil count < 500/mL); community-acquired pneumonia with bronchiectasis or cystic fibrosis; limb- or life-threatening diabetic foot or soft-tissue infection; or urinary tract infection.

Inappropriate: Penicillin allergy; treatment of a colonizing organism; treatment resulting from presumed contaminated cultures; or any indication not mentioned above.

Data Collection

Demographic variables such as age, gender, past medical history, and history of recent antimicrobial exposure were collected. The data were collected using the audit form. The appropriateness of carbapenem use, which can be either optimal, suboptimal, or inappropriate, was determined by the infectious disease team members who were independent of the study investigators.

Sampling: Universal sampling and non-probable sampling were employed.

## Results

Pre-intervention audit results

There were 20 subjects recruited into the study, comprised of 15 males (75%) and five females (25%). The mean age of the subjects was 48.9 years; the majority of them were Malay (12 patients, 60%), followed by Chinese (five patients, 25%), Indian (one patient, 5%), Iban (one patient, 5%), and Thai (one patient, 5%) (Table 1). Seventeen patients received meropenem, while three patients were treated with imipenem.

**Table 1:** Baseline characteristics of participants AML = acute myeloid leukemia.

Characteristic	Number of patients (%) (n = 20)
Gender	
Male	15 (75%)
Female	5 (25%)
Race	
Malay	12 (60%)
Chinese	5 (25%)
Indian	1 (5%)
Others	2 (10%)
Medical history	
Diabetes mellitus	10 (50%)
Cancer	1 (5%) - AML
Chronic kidney failure	11 (55%)
Recurrent urinary tract infection	1 (5%)
Chronic obstructive airway disease	1 (5%)
History of antimicrobial exposure in hospital within the past 3 months	
Cephalosporins	7
B-lactam and inhibitor combination	14
Quinolones	2
Macrolides	4
Clindamycin	1
Penicillin	2
Sulfa drugs	2

The audit revealed carbapenem prescription was appropriate in 13 cases (65%), suboptimal in five cases (25%), and inappropriate in two cases (10%) (Table 2). The most common indications for carbapenem order in the medical wards were hospital-acquired pneumonia (HAP)/ventilator-associated pneumonia (VAP) (eight cases) and urosepsis/renal abscess (five cases). Other indications included meningitis (two cases), continuous ambulatory peritoneal dialysis (CAPD) peritonitis (two cases), catheter-related bloodstream infection (two cases), and bacteremia (one case) (Table 3).

**Table 2:** Assessment of appropriateness of carbapenem therapy

Characteristic	Number of patients (%) (n = 20)
Appropriate	13 (65%)
Targeted therapy supported by cultures	10
Septic shock or severe infection with a history of infection in the past 6 months with a B-lactam-resistant organism	0
Septic shock or severe infection with a history of multiple antibiotics given or colonization with a B-lactam-resistant organism	0
Severe neutropenia with a history of multiple antibiotics given or colonization with a B-lactam-resistant organism	0
Concern for necrotizing pancreatitis	0
Persistent fever or hemodynamic instability in patients already receiving broad-spectrum B-lactam	3
Severe infection in a patient who has received broad-spectrum B-lactam therapy for a 5-day course in the past 30 days	0
As recommended or approved by infectious disease consult	0
Suboptimal	5 (25%)
Severe sepsis of unknown cause	2
Healthcare-associated pneumonia	2
Severe intra-abdominal infection	1
Severe neutropenia	0
Community-acquired pneumonia with bronchiectasis or cystic fibrosis	0
Limb- or life-threatening diabetic foot or soft tissue infection	0
Urinary tract infection	0
Inappropriate	2 (10%)
Penicillin allergy	1
Treatment of a colonizing organism	0
Treatment resulting from presumed contaminated culture	0
Any indication not mentioned above	1

**Table 3:** Sites of infection for patients treated with carbapenems

Site of infection	Suitability of therapy; number of cases			
	Appropriate	Suboptimal	Inappropriate	Overall (n = 20)
Central nervous system	0	1	1	2
Respiratory system	5	3	0	8
Gastrointestinal system	1	1	0	2
Genitourinary system	4	0	1	5
Blood	3	0	0	3

Post-intervention audit results

Twelve subjects were included in the post-intervention analysis, comprised of eight male and four female patients, and the mean age of the subjects was 58.6 years. Half of the subjects were Indian (six patients), followed by Malay (five patients) and Chinese (one patient) (Table 4). All patients were prescribed meropenem, as definitive therapy in seven patients and empirical therapy in five patients. The audit revealed carbapenem order was appropriate in 10 cases (83.3%) and suboptimal in two cases (16.7%) (Table 5).

**Table 4:** Baseline characteristics of participants

Characteristic	Number of patients (%) (n = 12)
Gender	
Male	8 (66.7%)
Female	4 (33.3%)
Race	
Malay	5 (41.7%)
Chinese	1 (8.3%)
Indian	6 (50%)
Others	0 (0%)
Medical history	
Diabetes mellitus	8 (66.7%)
Cancer	0 (0%)
Chronic kidney failure	7 (58.3%)
Recurrent urinary tract infection	0 (0%)
Chronic obstructive airway disease	1 (8.3%)
History of antimicrobial exposure in hospital within the past 3 months	
Cephalosporins	2
B-lactam and inhibitor combination	10
Quinolones	0
Macrolides	2
Clindamycin	0
Penicillin	0
Sulfa drugs	0

**Table 5:** Assessment of appropriateness of carbapenem therapy

Characteristic	Number of patients (%) (n = 12)
Appropriate	10 (83.3%)
Targeted therapy supported by cultures	6
Septic shock or severe infection with a history of infection in the past 6 months with a B-lactam-resistant organism	0
Septic shock or severe infection with a history of multiple antibiotics given or colonization with a B-lactam-resistant organism	0
Severe neutropenia with a history of multiple antibiotics given or colonization with a B-lactam-resistant organism	0
Concern for necrotizing pancreatitis	0
Persistent fever or hemodynamic instability in patients already receiving broad-spectrum B-lactam	2
Severe infection in a patient who has received broad-spectrum B-lactam therapy for a 5-day course in the past 30 days	0
As recommended or approved by infectious disease consult	2
Suboptimal	2 (16.7%)
Severe sepsis of unknown cause	0
Healthcare-associated pneumonia	1
Severe intra-abdominal infection	1
Severe neutropenia	0
Community-acquired pneumonia with bronchiectasis or cystic fibrosis	0
Limb- or life-threatening diabetic foot or soft tissue infection	0
Urinary tract infection	0
Inappropriate	0 (0%)
Penicillin allergy	0
Treatment of a colonizing organism	0
Treatment resulting from presumed contaminated culture	0
Any indication not mentioned above	0

HAP was the most common indication for carbapenem order (five cases), followed by bacteremia (four cases), meningitis (one case), perinephric collection (one case), and liver abscess (one case) (Table 6). Data comparison between pre- and post-intervention cohorts is summarized in Table 7.

**Table 6:** Sites of infection for patients treated with carbapenems

Site of infection	Suitability of therapy; number of cases			
	Appropriate	Suboptimal	Inappropriate	Overall (n = 12)
Central nervous system	1	0	0	1
Respiratory system	4	1	0	5
Gastrointestinal system	0	1	0	1
Genitourinary system	1	0	0	1
Blood	4	0	0	4

**Table 7:** Data comparison between pre- and post-intervention cohorts HAP = hospital-acquired pneumonia; VAP = ventilator-associated pneumonia; CAPD = continuous ambulatory peritoneal dialysis.

	Pre-intervention cohort (n = 20)	Post-intervention cohort (n = 12)
Mean age (years)	48.9	58.6
Major risk factors		
Diabetes mellitus	10/20 (50%)	8/12 (66.7%)
Chronic renal failure	11/20 (55%)	7/12 (58.3%)
Indication for carbapenem initiation	HAP/VAP (8 cases), urosepsis/renal abscess (5 cases), meningitis (2 cases), CAPD peritonitis (2 cases), catheter-related bloodstream infection (2 cases), bacteremia (1 case)	HAP (5 cases), bacteremia (4 cases), meningitis (1 case), perinephric collection (1 case), liver abscess (1 case)
Type of carbapenem used	Meropenem (17 cases), imipenem (3 cases)	Meropenem (12 cases)
Appropriateness of carbapenem order	13/20 = 65%	10/12 = 83.3%

## Discussion

This study showed that carbapenems prescribed in the medical wards at HSA were directed at a broad range of infections, ranging from lower respiratory tract infection, central nervous system infection, intra-abdominal infection, and genitourinary infection to bacteremia without evident foci. In our study, we found that the most frequent indication of carbapenem prescription was HAP/VAP.

A study at a tertiary-care teaching hospital in Brazil revealed that 42% of the 140 patients diagnosed with HAP were infected with MDR organisms and it is associated with higher mortality compared with non-MDR (45.8% vs. 38.3%). The use of broad-spectrum antibiotics within the last 10 days before the diagnosis of HAP was found to be an independent predictor of infection with MDR bacteria in non-ventilated patients with HAP [8]. The majority of our patients had prior broad-spectrum antimicrobial exposure, which was an important risk factor for infection with MDR organisms and hence the higher usage of carbapenems.

Despite the small sample size, we have shown that antimicrobial stewardship intervention increased the overall appropriateness of carbapenem prescription in the medical wards, from 65% (13/20) to 83.3% (10/12). We conducted multiple sessions of CME and also provided direct feedback to the clinicians to raise awareness of appropriate carbapenem treatment among the treating clinicians. The antimicrobial stewardship approach has been shown to successfully reduce unjustified antibiotic prescriptions and produce better patient outcomes, whereas inappropriate use of antimicrobial agents results in unnecessary medication exposure, worsening of infection, the emergence of resistance, and higher costs [9].

Antimicrobial stewardship programs have been shown to reduce rates of carbapenem-resistant organisms [10]. Antimicrobial stewardship interventions are more effective when implemented with infection control measures as a bundle of care, especially hand hygiene interventions [11]. Cipkoet al. demonstrated a reduction in carbapenemase-producing *Enterobacterales* isolates in conjunction with reduced carbapenem use. Following the implementation of the antimicrobial stewardship program, the average yearly consumption of carbapenems decreased by 20% [12].

The long-term carbapenems antimicrobial stewardship program had a positive impact on patient clinical outcomes and antibiotic resistance. An audit done in Spain demonstrated that the adequacy of carbapenems prescription improved from 49.7% to 80.9% after the implementation of long-term carbapenems antimicrobial stewardship program. Reduced and appropriate use of carbapenems may have a positive clinical and ecological impact, as well as decreased inpatient days, hospital-acquired MDR bloodstream infections, and candidemia, despite consumption of other antibiotics [13].

However, there are several limitations identified in our study. Firstly, the sample size in our study is small. We reviewed 20 patients for pre-intervention analysis and 12 patients for post-intervention analysis. The observational period was short with a limited number of patients being included in our study. Secondly, we did not collect data on patient outcomes including cure rate, mortality, length of hospitalization, and cost. Future research is therefore needed to determine whether this strategy can successfully achieve the three main objectives of antimicrobial stewardship programs, i.e., appropriate carbapenem prescribing, a decrease in carbapenem consumption, and an increase in the susceptibility of resistant pathogens, to ensure that ongoing programs are successful and long-lasting.

## Conclusions

Our study indicated that antimicrobial stewardship activities are effective in increasing the appropriateness of carbapenem use following the intervention phase. Antimicrobial activities that can be implemented include CME and direct feedback to clinicians to raise awareness of the proper use of carbapenem in clinical settings. The most common indication for which patients were prescribed carbapenem in our hospital was HAP/VAP.
